# Long-term outcomes in patients with COPD treated with non-invasive ventilation for acute hypercapnic respiratory failure

**DOI:** 10.1007/s11845-024-03690-w

**Published:** 2024-05-15

**Authors:** Shane O’Brien, Cara Gill, Niall Cograve, Mark Quinn, Ruairi J. Fahy

**Affiliations:** 1 Department of Respiratory Medicine, St. James’ Hospital, Dublin, Ireland; 2 School of Medicine, Trinity College Dublin, Dublin, Ireland

**Keywords:** Chronic Obstructive Pulmonary Disease, Home non-invasive ventilation, Mortality, Predictors of survival

## Abstract

**Purpose:**

Chronic Obstructive Lung Disease (COPD) remains a major cause of morbidity and mortality across the world. We evaluated survival over 9 years in a cohort of patients with COPD requiring acute inpatient non-invasive ventilation (NIV). We analyzed prognostic indices to evaluate if they were associated with mortality.

**Patients and methods:**

We performed a retrospective chart review of all patients who were admitted to St. James’s Hospital respiratory ward with COPD and acute hypercapnic respiratory failure who required NIV over a 12-month period and followed their outcomes over 9 years. We investigated the association between survival and potential prognostic variables using univariate analysis and multivariate Cox proportional hazards model. We evaluated the association between survival and the following parameters: age, gender, multiple admissions requiring NIV (> 1 admission in within 12 months of index presentation), home NIV use preadmission, initial arterial blood gas pH, days spent on NIV, serum albumin and serum albumin to serum CRP ratio at admission.

**Results:**

Ninety-nine patients with COPD and acute hypercapnic respiratory failure were identified over a 12-month period from January to December 2011. Survival at 1, 2, 5 and 9 years was 65% (*n* = 64), 42% (*n* = 42), 25% (*n* = 25) and 21% (*n* = 21), respectively. Increasing age (*p* value < 0.001) and a lower serum albumin (*p* value < 0.005) were associated with a higher mortality. There was a trend towards improved survival in the group who were treated with home NIV prior to admission compared to no NIV therapy at home but this did not reach statistical significance (Fig. 3, *p* value = 0.088).

**Conclusion:**

This study highlights the long-term mortality in patients with COPD admitted with hypercapnic respiratory failure requiring NIV and correlates with prior studies. Increasing age and lower serum albumin were associated with increased mortality. Home NIV may have a protective long-term survival benefit in patients with COPD who have been admitted for acute NIV.

## Introduction 

Despite declining rates of smoking in Europe and North America, Chronic Obstructive Lung Disease (COPD) remains a major cause of long-term morbidity and mortality with frequent hospitalizations adding to the cost of care [1, 2]. Patients admitted with acute exacerbations have a substantial hospital mortality rate [1]. Currently, the treatment of choice for hypercapnic respiratory failure is non-invasive mechanical ventilation (NIV) given its proven reduction in mortality [3]. Many of these studies were done in the ICU setting, though it is now common practice to manage these patients on a dedicated respiratory ward and little data is available on long-term survival from ward-based NIV services outside of a trial setting [1, 2]. Additionally, clinical or biological variables that could predict long-term survival may prove useful in a real-world setting. This is especially the case relating to home NIV, as recent evidence suggests that for those patients with persistent hypercapnia following an acute exacerbation of COPD, adding home non-invasive ventilation to home oxygen therapy prolonged the time to readmission or death within 12 months [4].

In this regard, we evaluated survival over a 9-year period in a cohort of patients, the majority of whom have COPD after an acute inpatient admission requiring NIV. We also analyzed prognostic indicators to evaluate if they were associated with mortality.

## Material and methods

St James’s Hospital is a large tertiary referral hospital in Dublin with a dedicated respiratory NIV unit since 2003. We performed a retrospective chart review of all patients who were admitted to St. James’s Hospital Respiratory ward with acute hypercapnic respiratory failure who required NIV over 12 months and followed their outcomes over 9 years. The primary endpoint was overall survival. Patients were treated with NIV if they met the selection criteria. At the time of study in our facility, this included an appropriate diagnosis of hypercapnic respiratory failure with potential reversibility requiring two of the following parameters:

1. Moderate to severe respiratory distress, tachypnoea, accessory muscle use
2. Arterial blood gas showing pH < 7.35, paC0_2_ > 6 kPa

Data retrospectively collected included demographics, comorbid medical illnesses, laboratory investigations, radiology and arterial blood gases in the acute setting, both initially and during treatment. We investigated the association between survival and potential prognostic variables using univariate analysis. The multivariate Cox proportional-hazards model was used to control for confounding factors. We adjusted the association between home NIV use, serum albumin and serum albumin to CRP ratio for age using the multivariate Cox proportional-hazards model. We evaluated the association between overall survival and the following parameters: age, gender, multiple admissions requiring NIV (> 1 admission within 12 months of index presentation), home NIV use pre-admission, initial arterial blood gas pH, days spent on NIV, serum albumin and serum albumin to serum CRP ratio at admission. Missing data was predominantly handled in the study design and with pairwise deletion of missing at random data. We had intended to assess the association between pulmonary function test parameters; however, due to an unacceptable level of missing PFT results, we decided to omit this from our analysis. Poisson regression was used to evaluate if any of the variables in our study were associated with the number of acute admissions requiring NIV. We attempted to minimize selection bias by including all patients who fulfilled the above selection criteria in the predefined time period. Patients with a diagnosis of obstructive sleep apnea or obesity hypoventilation syndrome were excluded as we feel this is a separate pathology which also causes hypercapnic respiratory failure. There is a ward-based system for capturing the diagnoses of each patient who is starting NIV. COPD was diagnosed by a respiratory physician in St. James’s Hospital and patients with a diagnosis of COPD treated with acute NIV were recorded in a ward-based database. We excluded four patients who had a diagnosis of obstructive sleep apnea. Potential sources of selection bias included patients who may have been too critically unwell to be deemed appropriate for a trial of NIV and therefore were not captured in this study. For instance, patients who have a ward-level ceiling of care, who in other circumstances require intubation, may have been treated with comfort measures rather than a trial of NIV depending on the specific circumstances.

This study involves human participants and was approved by the St James Hospital/Tallaght University Hospital Joint Research Ethics Committee, R&I number 706, submission number 168. This study complies with the declaration of Helsinki.

## Results

Ninety-nine patients were identified over 12 months from January to December 2011. Patient characteristics and results are summarized in Table 1.

**Table 1:** Patient characteristics and results

*n*	99
Age (mean (SD))	67.7 (15.9)
Sex,* M* (%)	45 (45.5)
Number of patients with COPD diagnosis	96 (96%)
Home NIV, *n* (%)	27 (27.3%)
1-year survival	65% (*n* = 64),
2-year survival	42% (*n* = 42)
5-year survival	25% (*n* = 25)
9-year survival	21%(*n* = 21)
Number of patients with > 1 admission for acute NIV within 12 months from index presentation (%)	21 (21.2%)
Time to death in days (mean (SD))	621 [ 74.0, 2163.5]
Days on NIV (median [IQR])	8 [5., 13.]
Death during index admission (%)	24 (24.2%)
Admission serum albumin g/L (mean (SD))	34.55 (5.33)
Admission ABG pH (mean (SD))	7.31 (0.08)
Admission pCO2 (mean)	8.00

There was an equal gender balance with female patients representing 55% (*n* = 54) of the cohort and 45% (*n* = 45) were male. The median age at admission was 67 years (range 29–93). There was no difference between males and females concerning survival, home NIV use, or death on index admission.

The median length of stay was 9 days (range 1–34). Twenty-seven percent of patients (*n* = 27) were on home NIV. Ninety-six percent of patients (*n* = 96) had a confirmed diagnosis of COPD.

**Table 2:** Results stratified by survival to discharge versus death on index admission

	Survived to discharge at index admission	Died at index admission	*p*
*n*	75	24	
Age (mean (SD))	63.6 (15.3)	80.2 (10.2)	< 0.01
Sex, *M* (%)	35 (46.7)	10 (41.7)	0.85
Time on NIV in days (median [IQR])	8.0 [5.0, 12.7]	8.50 [4.7, 14.0]	0.94
Home NIV, *N* (%)	23 (30.7)	4 (16.7)	0.28
Alb, g/L (mean (SD))	35.4 (5.0)	31.9 (5.5)	< 0.01
ph (mean (SD))	7.3 (0.08)	7.28 (0.07)	0.08

**Table 3:** Univariate analysis and Cox proportional hazard models

Variable	HR	95% Confidence intervals	*p*
Age	1.05	1.03–1.06	< 0.01
Sex	1.11	0.71–1.72	0.65
Days on NIV	0.99	0.96–1.01	0.63
Greater than one admission for acute NIV (%)	1.11	0.65–1.88	0.69
Home NIV	0.64	0.38–1.07	0.09
Admission serum albumin	0.96	0.92–0.99	0.03
Admission pH	0.12	0.003–3.44	0.21
CRP	1.01	0.99–1.01	0.17
Albumin to CRP ratio	0.97	0.95–0.99	0.03

Overall survival at 1, 2, 5 and 9 years was 65% (*n* = 64), 42% (*n* = 42), 25% (*n* = 25) and 21% (*n* = 21), respectively.

**Fig. 1:**
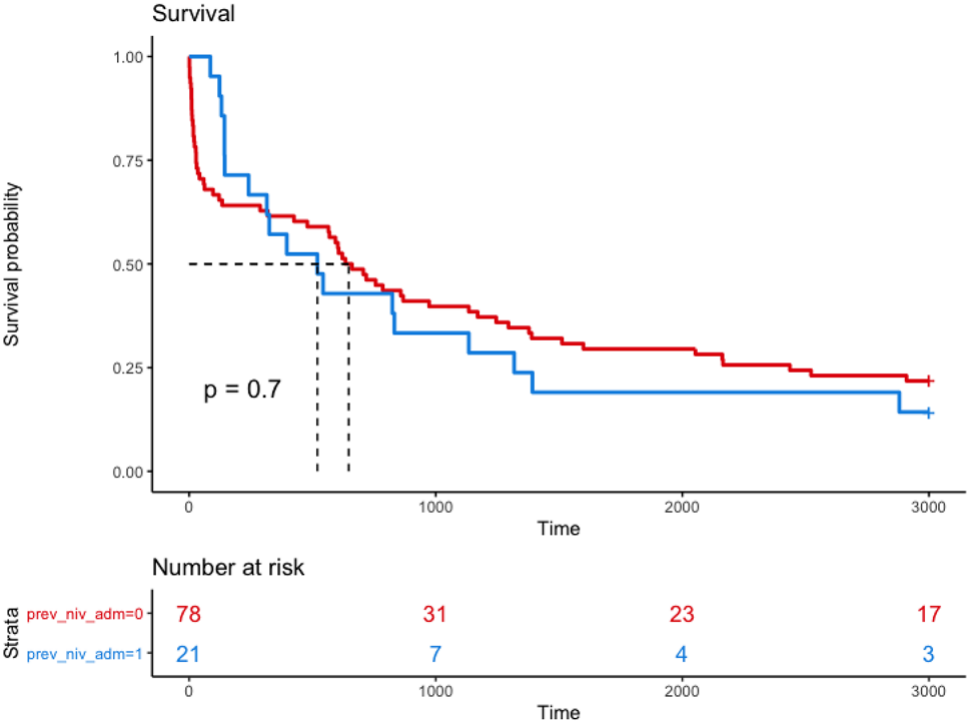
Kaplan-Meier survivor function stratified by multiple admissions (> 1 admission within 12 months of presentation) requiring non-invasive ventilation. Red line represents patients with just one admission in the first year and blue line represents patients with greater than one admission in the first year. Survival probability % on *y* axis and time in days on *x* axis

A total of 21.9% (*n* = 21) had recurrent admissions (> 1 within 12 months of index presentation requiring NIV (range between 2 and 5 admissions). Patients with multiple admissions for non-invasive ventilation (> 1 admission within 12 months of index presentation) had a lower 9-year survival than those requiring only one admission; however, there was no significant association between recurrent admissions and survival (*p* value = 0.7) (Fig. 1).

Survival at 1, 2 and 5 years for patients requiring > 1 admissions for NIV in the first year of study was 50% (*n* = 8), 37.5% (*n* = 6) and 25% (*n* = 4), respectively.

The small cohort of patients requiring > 2 admissions in the first year of the study (range 3–6 admissions) had a survival at 1, 2 and 5 years of 60% (*n* = 3), 20% (*n* = 1) and 0% (*n* = 0), respectively.

Poisson regression was utilized to evaluate if any of the variables in our study were associated with the number of admissions requiring NIV. There was an association with home NIV being protective with regard to the number of admissions (Table 4).

**Table 4:** Results stratified by whether patient was on home NIV before admission or not

	Not prescribed home NIV	Prescribed home NIV	*p*
*n*	72	27	
Age (mean (SD))	69.88 (15.5)	61.85 (15.6)	0.03
Gender, *M* (%)	33 (45.8)	12 (44.4)	1
Time to death in days (median [IQR])	579 [33.5, 1419.]	868 [320, 3000.]	0.07
Time on NIV in days (median [IQR])	7.00 [4.5, 13.0]	8.0 [5.0, 12.5]	0.42
Death on index presentation (%)	20 (27.8)	4 (14.8)	0.28
Greater than one admission for acute NIV	13 (18.1)	8 (29.6)	0.33
Alb, g/L (mean (SD))	34.4 (4.9)	34.9 (6.3)	0.64
ph (mean (SD))	7.3 (0.08)	7.3 (0.08)	0.40

**Fig. 2:**
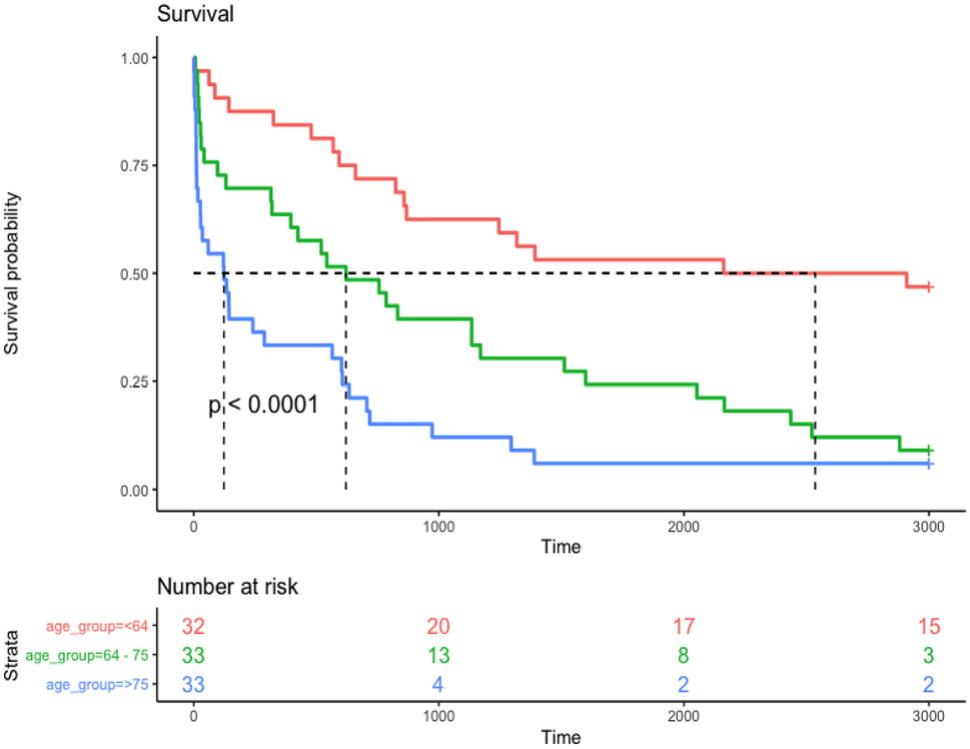
Kaplan-Meier survivor function stratified by age tertiles. Survival probability % on *y* axis and time in days on *x* axis. Red line represents patients less than 64 years old. The green line represents patients aged 64–75 years old. The blue line represents patients above 75 years old

Age was the greatest predictor of survival at index presentation, with a median age of 80.2 years amongst those who died compared to 63.6 years amongst those who survived to discharge (*p* < 0.001) (Table 2 and Fig. 2).

Serum albumin was considerably lower in the group that died at index presentation (mean serum albumin 32.0 g/L, *p* < 0.005) compared to those that survived to discharge (mean serum albumin 35.4 g/L, *p* < 0.005) (Table 2).

Using the Cox proportional hazard model, we showed that increasing age (age hazard ratio [HR], 1.05; confidence intervals [CI], 1.03–1.067; *p* value < 0.001) and lower serum albumin (albumin: HR, 0.96; CI 0.92–0.97; *p* value = 0.03) were associated with lower 9-year survival (Table 3).

There was no statistically significant association between sex, multiple admissions requiring NIV (> 1 admissions within 12 months of index presentation), initial arterial blood gas pH, days on NIV, or preexisting home NIV use and 9-year survival. When adjusted for age using multivariate analysis, albumin, albumin to CRP ratio and home NIV use did not produce a significant *p* value with respect to predicting survival.

**Fig. 3:**
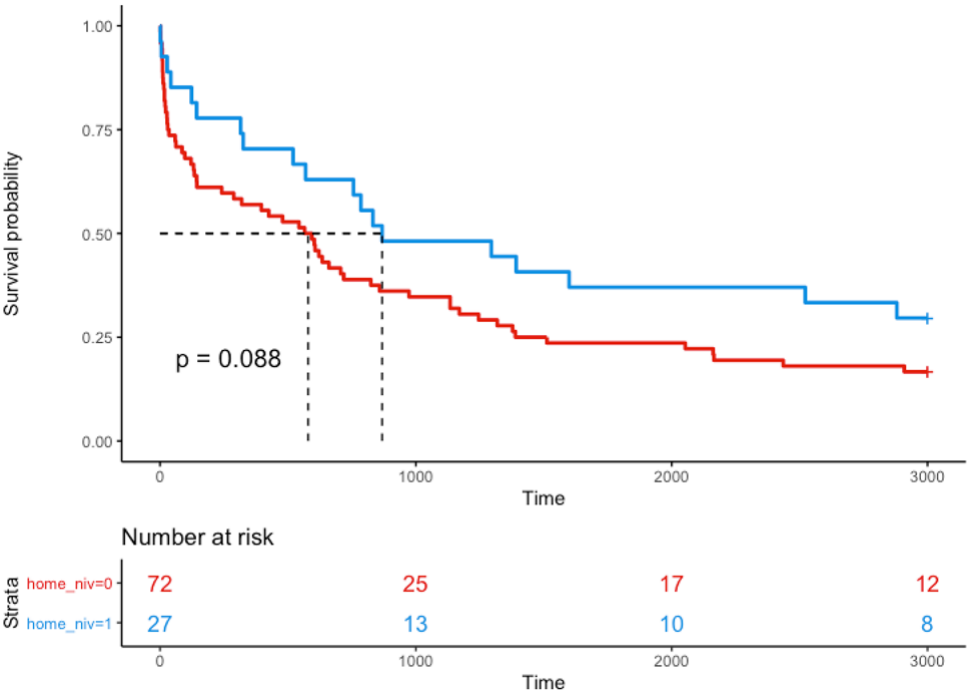
Kaplan-Meier survivor function stratified by the use of non-invasive ventilation (NIV) at home before admission. Red line represents patients not on home NIV and blue line represents patients on home NIV. Survival probability % on *y* axis and time in days on *x* axis

There was a trend towards improved 9-year survival in the group who had pre-existing home NIV before admission compared to no NIV therapy at home (Fig. 3, *p* value = 0.088). Although there is a trend toward a positive finding here, patients on home NIV were younger (mean age, 61.85 years versus 69.88 years) (Table 3).

On reviewing data 9 years on from the index admission, 21 patients (21%) were still alive and 8 of these are on home NIV, suggesting a trend toward NIV having a protective effect (*p* = 0.11) (Table 4).

## Discussion

The early use of NIV for patients with COPD and hypercapnic respiratory failure in the general ward setting leads to a improvement of physiological variables, a reduction in the need for invasive mechanical ventilation and a reduction in in hospital mortality [5].

The 1-, 2-, 5- and 9-year survival figures in our study of patients treated with NIV were 65% (*n* = 64), 42% (*n* = 42), 25% (*n* = 25) and 21% (*n* = 21), respectively, which were similar to what has been reported in the literature to date.

Cabrini et al. reported a 1-year survival of 66% in patients with COPD receiving ward-based non-invasive ventilation for acute respiratory failure [6]. Titlestad et al. reported a 5-year survival of 23% in patients receiving NIV for the first time after being admitted acutely to an acute medical ward with later transfer to a respiratory ward with respiratory failure and a diagnosis of COPD [7].

Chung et al. found that the long-term survival of patients surviving the first episode of type II respiratory failure requiring non-invasive ventilation (NIV) at 1, 2 and 5 years was 72%, 52% and 26%, respectively [8]. In their study, only advanced age (*p* = 0.04), BMI (*p* = 0.014) and prior domiciliary oxygen use (*p* = 0.03) correlated with death within 5 years. Physiological measures of the severity of respiratory failure at presentation did not predict survival and this is in keeping with our findings in this study. In patients admitted to hospital with an exacerbation of COPD, Sprooten and colleagues identified advanced age, NIV use more than 8 days and unsuccessful response to NIV as clinical important independent predictors for long-term mortality [9]. Age was the greatest predictor of survival in our study, and we did not find an association between the severity of acidosis or time spent on NIV at presentation and survival. In our study, lower serum albumin was associated with a worse survival. It is known that serum albumin reflects nutritional status which may account for the association. However, other stressors can reduce serum albumin [10]. Serum albumin is depressed during inflammation, and thus, it is intuitive that patients presenting with exacerbations of COPD or infection may have lower serum albumin [11]. In our study, lower serum albumin was associated with worse survival overall and this may be related to the patient’s nutritional status and/or levels of inflammation [12].

There was no significant association with recurrent admissions in the first year and subsequent survival over 9 years in our study. This may be due to survivor bias; one needs to be alive to have a subsequent admission, and therefore, those who died early in the study did not contribute to the mortality associated with recurrent admissions. Also, the number of patients with multiple admissions was relatively small (*n* = 21) and this too may have impacted the statistical power.

Regarding the use of home NIV in patients with COPD and hypercapnia, evidence is accumulating from both metanalysis and randomized controlled clinical trials that home NIV may reduce admissions and improve survival. Wilson and colleagues performed a meta-analysis of patients with COPD and hypercapnia and found that home NIV, compared with no device, was associated with lower risk of mortality (22.31% vs 28.57%; risk difference [RD], −5.53% [95% CI, −10.29 to −0.76%]; odds ratio [OR], 0.66 [95% CI, 0.51–0.87]; *p* = .003; 13 studies; 1423 patients; strength of evidence [SOE], moderate), hospital admissions and intubation [13]. The American Thoracic Society guidelines now suggest the use of nocturnal NIV in addition to usual care for patients with chronic stable hypercapnic COPD [14]. Our data show a trend towards improved survival over 9-year follow-up in patients using home NIV, and this adds to the growing body of evidence for the use of home NIV in patients with COPD and hypercapnia. This study was a retrospective, single-centre observational study without a comparator group and results need to be taken within this context; however, despite this, we feel that it adds value to the field. Survival in COPD is poor overall. Five-year survival in patients with COPD was 79% compared to 95% survival in controls with unobstructed pulmonary function tests matched for age, comorbidities and smoking history [15]. In a recent study which aimed to determine whether long-term NIV (LT-NIV) initiated after an admission with acute hypercapnic respiratory failure (AHRF) can affect survival and admission rate in COPD patients, time to readmission with AHRF or death within 12 months was numerically smaller in the LT-NIV group, however did not reach significance [16]. The poor 9-year survival in our cohort once likely reflects the high mortality associated with exacerbations causing hypercapnic respiratory failure in patients with COPD. The cohort of patients with more exacerbations leading to hypercapnic respiratory failure had a lower survival; however, the numbers in this study were likely too small to produce a significant result.

## Conclusion

This study highlights the long-term mortality in patients with COPD admitted with hypercapnic respiratory failure requiring NIV and correlates with prior studies. Increasing age and lower serum albumin are associated with increased mortality. Home NIV may have a protective long-term survival benefit in patients with COPD who have been admitted for acute NIV. Serum albumin could also be a potentially useful predictive biomarker of survival in this context.

## Data Availability

Data is available from the corresponding author upon request.

## Declarations

### Patient and public involvement statement

The main outcome of this study is survival and predictors of survival, and we feel this is patient-centred. Patients were not directly involved in the design of this study.
